# Prognostic significance of serum albumin in patients with stable coronary artery disease treated by percutaneous coronary intervention

**DOI:** 10.1371/journal.pone.0219044

**Published:** 2019-07-03

**Authors:** Sho Suzuki, Naoto Hashizume, Yusuke Kanzaki, Takuya Maruyama, Ayako Kozuka, Kumiko Yahikozawa

**Affiliations:** Department of Cardiovascular Medicine, Minaminagano Medical Center, Shinonoi General Hospital, Nagano, Japan; Azienda Ospedaliero Universitaria Careggi, ITALY

## Abstract

**Background:**

Stable coronary artery disease (CAD) is known to have an increased risk of cardiovascular events. Serum albumin (Alb) is reported as a useful risk-stratification tool in cardiovascular diseases such as acute coronary syndrome or heart failure. However, the association between Alb and stable CAD is unclear. Thus, we aimed to investigate the prognostic significance of Alb in patients with stable CAD.

**Methods and results:**

We analyzed the data of all patients admitted to Shinonoi General Hospital between October 2014 and October 2017 for newly diagnosed stable CAD, treated via elective percutaneous coronary intervention, with the exception of old myocardial infarction. We collected data, including Alb, at admission. The primary endpoint was major adverse cardiac events (MACE; defined as all-cause death, non-fatal myocardial infarction, non-fatal stroke). In 204 enrolled patients (median age, 73 years), during a median follow-up of 783 days, 28 experienced MACE. Alb was significantly lower in patients with MACE than in those without (p<0.001). In Kaplan-Meier analysis, low Alb predicted worse prognosis in MACE (p<0.001). In multivariate Cox regression analysis, low Alb levels independently predicted MACE (p<0.001) after adjusting for age and sex (HR 4.128 [95% CI 1.632–10.440], p = 0.003), or, age and C-reactive protein (HR 3.373 [95% CI 1.289–8.828], p = 0.013).

**Conclusions:**

Low Alb levels predicted MACE in patients with stable CAD.

## Introduction

Stable coronary artery disease (CAD) is known to have an increased risk of cardiovascular events [1]. Several biomarkers such as cardiac troponin, hemoglobin, mean platelet volume, and C-reactive protein (CRP) are reported as predictors of adverse events in stable CAD patients [2–5].

Serum albumin (Alb) is the most abundant protein in plasma, representing the main determinant of plasma oncotic pressure and the main modulator of fluid distribution between the body compartments [6]. Alb is associated with coronary heart disease [7], with possible mechanisms, including responses to inflammation [8]. Moreover, the association between low Alb and increased risks of cardiovascular disease and heart failure is reported in several studies [9, 10]. In acute coronary syndrome, low Alb was associated with increased severity of coronary lesions, in-hospital mortality, heart failure, and all-cause death [11–13]. A recent study investigated the association between low Alb concentration and adverse cardiovascular events in stable coronary heart disease including old myocardial infarction, previous coronary artery disease, and heart failure [14]. However, the prognostic significance of low Alb level at admission in patients with newly diagnosed CAD who underwent percutaneous coronary intervention (PCI) is not well established. Therefore, we aimed to investigate the association between low Alb levels and adverse events in a retrospective cohort of patients with newly diagnosed stable CAD.

## Materials and methods

### Study population

This was a retrospective, single-center cohort study. The cohort included patients admitted to Shinonoi General Hospital between October 2014 and October 2017 for newly diagnosed stable CAD with the exception of old myocardial infarction, treated via elective PCI. Patients with malignancy were excluded. All patients were enrolled after the approval of the Shinonoi General Hospital Ethics Committee, and after written informed consent was obtained. The collected data included clinical characteristics, medical history, major risk factors for CAD, comorbidities, laboratory, electrocardiography, echocardiography, and angiographic data, discharge medications, and post-discharge follow-up findings. All data were fully anonymized before access. The investigation is consistent with the principles outlined in the Declaration of Helsinki.

We measured Alb at admission using the bromocresol purple assay and the LABOSPECT 008 analyzer (Hitachi Ltd, Tokyo, Japan). The measurement range of the Alb assay was 1.0–8.0 g/dL with a coefficient of variation of 5%. The minimum detection limit was 0.1 g/dl.

### Definitions and endpoints

Diagnosis of old myocardial infarction was made by the treating clinicians using all available data, including symptoms, laboratory findings, electrocardiograms, echocardiograms, and coronary angiograms. Stable CAD was defined as angiographic evidence of ≥90% epicardial coronary artery stenosis or angiographic evidence of ≥75% epicardial coronary artery stenosis with either a symptom of chest pain induced by exercise or evidence of stress-induced ischemia via any clinical stress-testing modality. Aspirin and thienopyridines were administered before the procedure. Coronary angiography and PCI were performed using standard protocols and guidelines.

### Follow-up

The primary outcome was major adverse cardiac events (MACE; defined as all-cause mortality, non-fatal myocardial infarction, and non-fatal stroke) and the secondary outcome was cardiovascular events (CV events; defined as cardiovascular death, non-fatal myocardial infarction, and non-fatal stroke). Incidents were validated by chart review.

### Statistical analysis

Continuous variables are summarized as mean ± standard deviation, if normally distributed and as median [interquartile range], if non-normally distributed. Normality was assessed by the Shapiro-Wilk W-test. Comparisons of baseline characteristics were conducted with a contingency table. Pearson’s χ ^2^ test was used for categorical variables, the t-test was used for normally distributed continuous variables, and the Wilcoxon or Mann-Whitney test was used for non-normally distributed continuous variables. Spearman’s rank correlation method was used as a nonparametric measure of the association between Alb and clinical indices. Patients were then divided into 2 groups according to the median Alb level: the high Alb group (Alb ≥4.0 g/dL, n = 89), and the low Alb group (Alb <4.0 g/dL, n = 115). Kaplan-Meier survival plots were calculated from baseline to the time of MACE and compared using the log-rank test. Cox proportional-hazards analysis was used to evaluate the independent prognostic utility of Alb. The covariates used were age, sex, body mass index (BMI), hemoglobin, estimated glomerular filtration rate (eGFR), and CRP. Because the study included only a small number of patients, power calculation was performed. In addition, to evaluate the prognostic significance of CRP/Alb ratio in this study, a similar analysis was repeated using the CRP/Alb ratio. A p-value of <0.05 was considered statistically significant. All statistical analyses were performed using SPSS Statistics for Windows, Version 25 (IBM Corp., Armonk, NY, US).

## Results

### Study population

We enrolled 204 patients (mean age, 72 years; male, 69%). The baseline characteristics are shown in Table 1. Patients who developed MACE compared to those who did not were older and had lower Alb (3.5 [3.0–3.8] g/dL vs. 4.1 [3.8–4.4] g/dL, p <0.001), hemoglobin, BMI, total cholesterol, and low density lipoprotein cholesterol. CRP and CRP/Alb ratio were higher in patients who developed adverse events than in those who did not. However, no significant correlations were detected between Alb and these clinical indices (Table 2). The baseline lesional characteristics are shown in Table 3.

**Table 1:** Baseline characteristics.

Variable	Overall Population	MACE		p-value
	(n = 204)	YES (n = 28)	NO (n = 176)	
Age (years)	72 [67–79]	77 [69–87]	72 [65–79]	0.004
Male sex, n (%)	142 (69)	19 (68)	123 (70)	0.828
BMI	23.4 [21.0–25.7]	21.3 [18.6–23.3]	23.8 [21.4–25.9]	<0.001
Systolic blood pressure (mmHg)	136 [123–146]	136 [124–149]	137 [123–147]	0.878
Diastolic blood pressure (mmHg)	77 [71–86]	77 [61–90]	76 [70–85]	0.674
Hypertension, n (%)	151 (74)	21 (75)	130 (74)	0.899
Dyslipidemia, n (%)	104 (51)	10 (36)	87 (53)	0.082
Diabetes mellitus, n (%)	73 (36)	8 (40)	65 (37)	0.391
Atrial fibrillation, n (%)	26 (13)	6 (27)	20 (11)	0.122
OCI, n (%)	35 (17)	8 (40)	27 (15)	0.078
PAD, n (%)	53 (26)	13 (46)	40 (23)	0.008
Past smoker, n (%)	101 (49)	9 (32)	92 (52)	0.051
LVEF (%)	66 [63–68]	67 [61–68]	66 [62–68]	0.949
**Medication**				
Aspirin, n (%)	203 (99)	27 (99)	175 (99)	0.256
Thienopiridines, n (%)	201 (98)	27 (99)	173 (98)	0.449
Warfarin, n (%)	5 (2.4)	2 (7)	3 (2)	0.14
DOAC, n (%)	21 (10)	4 (14)	17 (10)	0.32
Statin, n (%)	111 (54)	12 (43)	99 (56)	0.186
Ezetimibe, n (%)	3 (1.5)	0 (0)	3 (2)	0.641
PPI, n (%)	135 (66)	13 (46)	121 (69)	0.021
ACE-Is, n (%)	19 (9)	3 (11)	16 (9)	0.501
ARBs, n (%)	88 (43)	14 (50)	74 (42)	0.43
Beta-blockers, n (%)	55 (27)	6 (21)	49 (28)	0.478
MRAs, n (%)	11 (5.4)	2 (7)	9 (5)	0.462
**Laboratory data**				
Hb (g/dL)	14.1 [12.1–15.2]	12.0 [10.5–14.0]	14.0 [12.6–15.2]	<0.001
Alb (g/dL)	4.0 [3.6–4.3]	3.5 [3.0–3.8]	4.1 [3.8–4.4]	<0.001
eGFR (mL/min/1.73m2)	63 [53–72]	58 [35–74]	65 [54–76]	0.127
AST (U/L)	23 [18–29]	22 [18–29]	23 [18–29]	0.797
ALT (U/L)	18 [14–26]	17 [9–22]	18 [14–27]	0.12
T-Chol (mg/dL)	184 [168–208]	167 [140–193]	186 [168–209]	0.014
HDL-Chol (mg/dL)	48 [41–57]	48 [38–62]	49 [41–57]	0.54
LDL-Chol (mg/dL)	109 [88–129]	92 [76–115]	110 [92–131]	0.015
Triglycerides (mg/dL)	103 [76–154]	93 [61–181]	118 [84–157]	0.35
CRP (mg/dL)	0.09 [0.04–0.35]	0.41 [0.06–1.03]	0.11 [0.04–0.25]	0.004
CRP/Alb × 100	2.9 [1.1–8.9]	11.8 [2.7–42.4]	2.5 [1.0–7.0]	0.001
HbA1c (%)	5.9 [5.7–6.4]	5.9 [5.5–7.0]	6.0 [5.7–6.7]	0.596

Values are presented as median and interquartile range, or n (%). Abbreviations: ACE-I = angiotensin-converting enzyme inhibitor; Alb = serum albumin; ARB = angiotensin-receptor blocker; BMI = body mass index; CRP = C-reactive protein; DOAC = direct oral anticoagulants; eGFR = estimated glomerular filtration rate; Hb = hemoglobin; HbA1c = hemoglobin A1c; HDL Chol = high-density lipoprotein cholesterol; LDL Chol = low density lipoprotein cholesterol; LVEF = left ventricular ejection fraction; MACE = major adverse cardiac events; MRA = mineralocorticoid receptor antagonist; OCI = old cerebral infarction; PAD = peripheral artery disease; PPI = proton pump inhibitor; T Chol = total cholesterol.

**Table 2:** Univariate Spearman’s rank correlations between serum albumin and clinical indices.

Variable	Spearman’s Rank	p-value
Age (years)	-0.368	<0.001
Sex	0.092	0.191
BMI	0.311	<0.001
Hb (g/dL)	0.604	<0.001
eGFR (ml/min/1.73m2)	0.375	<0.001
T Chol (mg/dL)	0.393	<0.001
LDL Chol (mg/dL)	0.394	<0.001
CRP (mg/dL)	-0.452	<0.001

Abbreviations as in Table 1.

**Table 3:** Lesional characteristics.

	Overall population	Alb <4.0 g/dL	Alb ≥4.0 g/dL	p-value
	(n = 204)	(n = 89)	(n = 115)	
Multivessel disease (%)	53 (26)	26 (29)	27 (24)	0.354
LMT lesions (%)	13 (6)	8 (9)	5 (4)	0.178
Calcified lesions (%)	29 (14)	20 (23)	9 (8)	0.003
Ostial lesions (%)	30 (15)	15 (17)	15 (13)	0.446
Bifurcation lesions (%)	102 (50)	45 (51)	57 (50)	0.888
CTO lesions (%)	12 (6)	3 (3)	9 (8)	0.18
DES use (%)	193 (95)	83 (93)	110 (96)	0.453
BMS use (%)	11 (5)	6 (7)	5 (4)	0.453

Values are presented as median [interquartile range], or n (%). Abbreviations: Alb = serum albumin; BMS = bare metal stent; CTO = chronic total occlusion; DES = drug-eluting stent; LMT = left main trunk.

### The prognostic impact of serum albumin

**Fig 1:**
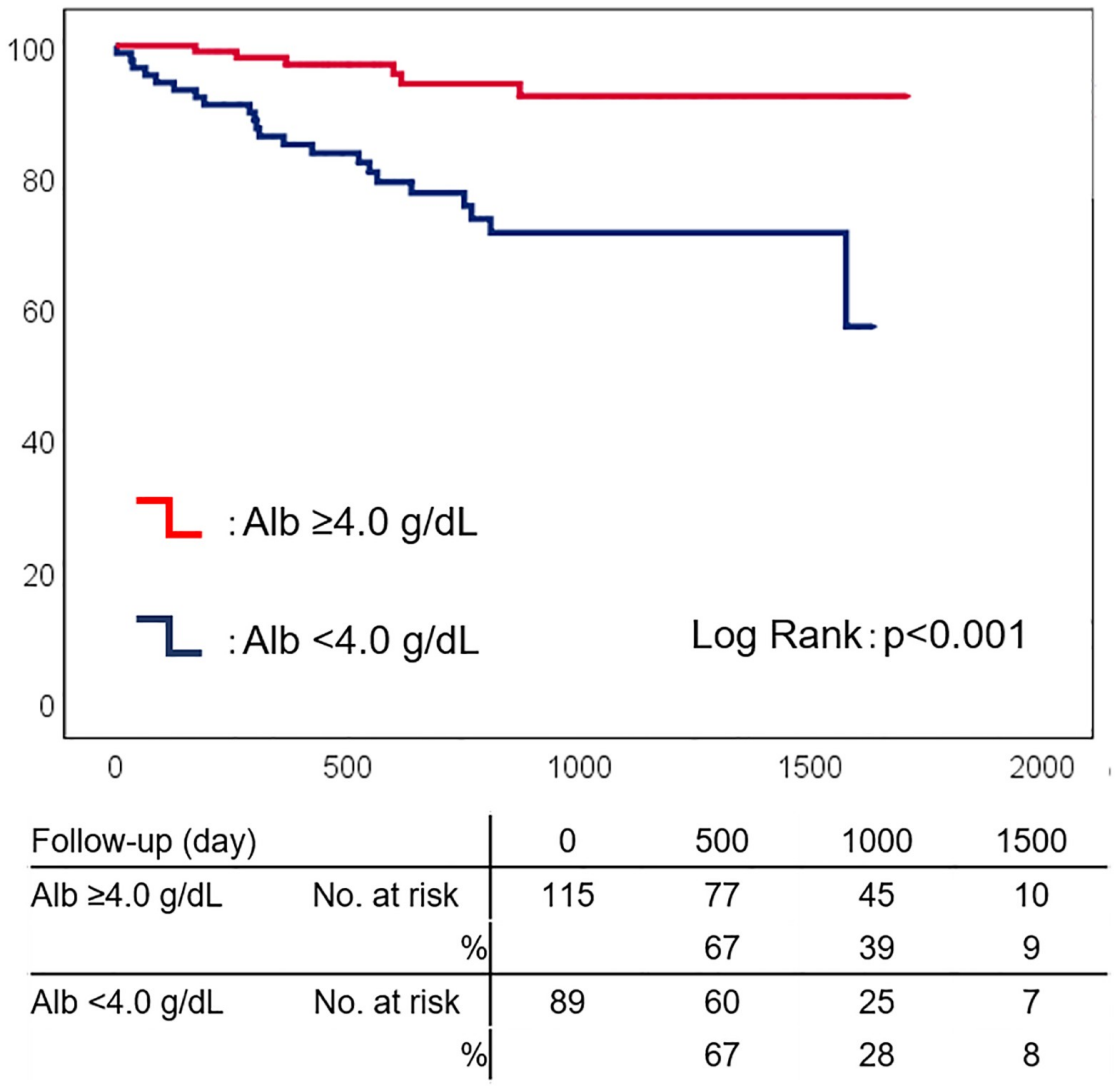
Kaplan-Meier analysis of serum albumin in patients with stable coronary artery disease. Low serum albumin (Alb, <4.0 g/dL) predicted major adverse cardiac events (blue line). Red line, Alb ≥4.0 g/dL.

During a median follow-up of 742 days [interquartile range: 428–1122], 28/204 (13.7%) patients experienced MACE (all-cause death, 18; non-fatal myocardial infarction, 3; non-fatal stroke, 11). Low Alb levels were related to an increased risk of MACE (Low Alb group: 24.7% [22/89] vs High Alb group: 5.2% [6/115], p<0.001). In Kaplan-Meier analysis, Alb <4.0 g/dL predicted MACE (Fig 1). In multivariate Cox proportional hazards analysis, Alb <4.0 g/dL predicted MACE after adjustment for age, sex, BMI, hemoglobin, eGFR, and CRP (Table 4). After analysis, the power of the study was 98.5%. In the secondary endpoint, 19/204 (9.3%) patients experienced CV events (cardiac death, 6; non-fatal myocardial infarction, 3; non-fatal stroke, 11). In multivariate Cox proportional hazards analysis, Alb <4.0 g/dL also predicted CV events after adjusting for age (HR 3.344 [95% CI 1.172–9.545], p = 0.024), sex (HR 3.879 [95% CI 1.396–10.781], p = 0.009), and CRP (HR 3.053 [95% CI 1.038–8.977], p = 0.043). Regarding the analysis in CRP/Alb ratio, patients were divided into 2 groups according to the median CRP/Alb ratio level: the high CRP/Alb ratio group (CRP/Alb ratio ≥2.9, n = 98), and the low CRP/Alb ratio group (CRP/Alb ratio <2.9, n = 98). CRP/Alb ratio also predicted MACE on multivariate Cox proportional hazards analysis after adjusting for age and sex (HR 2.708 [95% CI 1.134–6.466], p = 0.025).

**Table 4:** Multivariable Cox proportional hazards analysis of major adverse cardiac events.

Variable	HR (95% CI)	p-value
Albumin adjusted for		
Age, Sex	4.128 (1.632–10.440)	0.003
Age, BMI	3.332 (1.288–8.615)	0.013
Age, Hb	2.76 (1.033–7.372)	0.043
Age, eGFR	4.093 (1.595–10.503)	0.003
Age, CRP	3.373 (1.289–8.828)	0.013

Abbreviations: BMI = body mass index; CI = Confidence interval; CRP = C-reactive protein; eGFR = estimated glomerular filtration rate; Hb = hemoglobin; HR = Hazard ratio.

## Discussion

In this study, we identified a significant association between low Alb at admission and MACE in patients hospitalized with newly diagnosed stable CAD. This association was independent of other risk predictors. Previous studies reported an association between low Alb and increased risks of CV events in the general population [9, 10, 15–17]. Moreover, the prognostic value of low Alb has been reported in acute coronary syndrome and stable coronary heart disease including old myocardial infarction and heart failure [11–14]. However, to the best of our knowledge, no prior study has investigated the use of Alb in patients with newly diagnosed stable CAD treated via successful PCI. Although several biomarkers are reported as a predictor of adverse events in stable CAD, risk-stratification tools are still lacking [18]. In this study, we demonstrated the prognostic value of Alb in predicting MACE and CV events. Our findings have important clinical implications and suggest that Alb is a useful risk-stratification tool in cases of newly diagnosed stable CAD. Moreover, our study indicates that Alb measurement at admission could identify patients who require aggressive therapy and close outpatient follow-up.

Plasma concentration of albumin is controlled by several processes, including the rate of albumin synthesis, the fractional catabolic rate, albumin distribution between the vascular and extravascular compartments, and exogenous albumin loss [19–21]. Low Alb concentration is caused by either the decrease of albumin synthesis, plasma volume expansion, or direct loss of protein from the body. The rate of albumin synthesis is affected by nutritional intake and systemic inflammation [19]. In previous studies, which reported the association between low Alb and adverse events in heart failure, inflammation was suggested as the underlying etiology of low Alb [20]. Elevated levels of inflammatory mediators, including CRP, have been identified as a predictor of poor prognosis in chronic and acute heart failure, which supports the pathophysiologic theory [22–24]. Recent studies have reported the prognostic significance of CRP/Alb ratio in patients with ST elevation myocardial infarction or stable CAD, and suggested that CRP/Alb ratio indicates higher inflammation caused by atherosclerosis [25, 26]. In our study, CRP was significantly higher in patients who developed MACE compared to those who did not, and CRP/Alb ratio independently predicted MACE. Findings from our study suggest that low Alb in stable CAD is caused by systemic inflammation in atherosclerosis. There is evidence that low-grade chronic inflammation and underlying cellular and molecular mechanisms are present in atherosclerosis [27–29]. Therefore, it may be possible that patients who develop MACE in stable CAD have more severe atherogenesis-related inflammation. However, this hypothesis is only speculative, and further studies are needed.

Certain treatments that reduce coronary risks, such as dyslipidemia, hypertension, diabetes mellitus, obesity, and smoking limit inflammation [27]. Moreover, there is strong evidence that statin therapy can reduce inflammation in atherosclerosis [27, 30, 31]. Stable CAD patients with low Alb should receive medical tests (e.g. urine analysis or computed tomography) to identify the reason of Alb decrease, and at the same time, receive aggressive therapy to reduce coronary risks. Low density lipoprotein cholesterol should be severely controlled to reduce atherosclerosis. If nutritional intake is lacking, the Nutrition Support Team may support their diet. Interdisciplinary approach should be considered in these patients.

Our study had several limitations. First, we included a small number of patients taken from a single center. Furthermore, the median age of this study was relatively high. The study cohort represents only a limited part of society, and further research involving a large cohort is necessary to verify our findings. Second, only one Alb measurement was available in each case, and serial Alb levels were not evaluated. Third, survival status was ascertained by chart review alone and the median follow-up period was short. Finally, although the transaminase levels were normal, this study did not exclude patients with hepatic failure.

## Conclusions

Low Alb was independently associated with adverse clinical events in patients with newly diagnosed stable CAD. Our findings suggest that Alb may be a useful risk-stratification tool in this population. Further studies are needed to identify the mechanism of Alb decrease in these patients.

## References

[1] Steg PG, Bhatt DL, Wilson PW, D’Agostino R Sr, Ohman EM, Röther J, et al One-year cardiovascular event rates in outpatients with atherothrombosis. JAMA. 2007;297:1197–1206. 10.1001/jama.297.11.1197 17374814

[2] Vavik V, Pedersen EKR, Svingen GFT, Tell GS, Schartum-Hansen H, Aakre KM, et al Usefulness of higher levels of cardiac troponin t in patients with stable angina pectoris to predict risk of acute myocardial infarction. Am J Cardiol. 2018;122:1142–7. 10.1016/j.amjcard.2018.06.027 30146101

[3] Kitai Y, Ozasa N, Morimoto T, Bao B, Furukawa Y, Nakagawa Y, et al Prognostic implications of anemia with or without chronic kidney disease in patients undergoing elective percutaneous coronary intervention. Int J Cardiol. 2013;168:5221–8. 10.1016/j.ijcard.2013.08.029 23998544

[4] Jiang P, Song Y, Xu JJ, Wang HH, Jiang L, Zhao W, et al Two-year prognostic value of mean platelet volume in patients with diabetes and stable coronary artery disease undergoing elective percutaneous coronary intervention. Cardiol. J; 10.5603/CJ.a2018.0071 30009376 PMC8086647

[5] Vasan RS. Biomarkers of cardiovascular disease: molecular basis and practical considerations. Circulation. 2006;113:2235–62. 10.1161/CIRCULATIONAHA.104.482570 16702488

[6] Fanali G, di Masi A, Trezza V, Marino M, Fasano M, Ascenzi P. Human serum albumin: from bench to bedside. Mol Aspects Med. 2012;33:209–90. 10.1016/j.mam.2011.12.002 22230555

[7] Danesh J, Collins R, Appleby P, Peto R. Association of fibrinogen, C-reactive protein, albumin, or leukocyte count with coronary heart disease: meta-analyses of prospective studies. JAMA. 1998;279:1477–82. 9600484 10.1001/jama.279.18.1477

[8] Gabay C, Kushner I. Acute-phase proteins and other systemic responses to inflammation. N Engl J Med. 1999;340:448–54. 10.1056/NEJM199902113400607 9971870

[9] Schalk BW, Visser M, Bremmer MA, Penninx BW, Bouter LM, Deeg DJ. Change of serum albumin and risk of cardiovascular disease and all-cause mortality: Longitudinal Aging Study Amsterdam. Am J Epidemiol. 2006;164:969–77. 10.1093/aje/kwj312 16980573

[10] Gopal DM, Kalogeropoulos AP, Georgiopoulou VV, Tang WW, Methvin A, Smith AL, et al Serum albumin concentration and heart failure risk the Health, Aging, and Body Composition Study. Am Heart J. 2010;160:279–85. 10.1016/j.ahj.2010.05.022 20691833 PMC2919495

[11] Kurtul A, Murat SN, Yarilioglues M, Duran M, Ocek AH, Koseoglu C, et al Usefulness of serum albumin concentration to predict high coronary SYNTAX score and in-hospital mortality in patients with acute coronary syndrome. Angiology. 2016;67:34–40. 10.1177/0003319715575220 25783433

[12] González-Pacheco H, Amezcua-Guerra LM, Sandoval J, Martinez-Sánchez, Ortiz-León XA, Peña-Cabral MA, et al Prognostic implications of serum albumin levels in patients with acute coronary syndromes. Am J Cardiol. 2017;119:951–8. 10.1016/j.amjcard.2016.11.054 28160977

[13] Oduncu V, Erkol A, Karabay CY, Kurt M, Akgün T, Bulut M, et al The prognostic value of serum albumin levels on admission in patients with acute ST segment elevation myocardial infarction undergoing a primary percutaneous coronary intervention. Coron Artery Dis. 2013;24:88–94. 10.1097/MCA.0b013e32835c46fd 23249632

[14] Chien SC, Chen CY, Leu HB, Su CH, Yin WH, Tseng WK, et al Association of low serum albumin concentration and adverse cardiovascular events in stable heart disease. Int J Cardiol. 2017;241:1–5. 10.1016/j.ijcard.2017.04.003 28413113

[15] Phillips A, Shaper AG, Whincup PH. Association between serum albumin and mortality from cardiovascular disease, cancer, and other causes. Lancet. 1989;2:1434–6. 10.1016/s0140-6736(89)92042-4 2574367

[16] Gillum RF, Makuc DM. Serum albumin, coronary heart disease, and death. Am Heart J. 1992;123:507–13. 1736588 10.1016/0002-8703(92)90667-k

[17] Kuller LH, Eichner JE, Orchard TJ, Grandits GA, McCallum L, Tracy RP. The relation between serum albumin levels and risk of coronary heart disease in the multiple risk factor intervention trial. Am J Epidemiol. 1991;134:1266–77. 10.1093/oxfordjournals.aje.a116030 1755441

[18] Hemingway H, Philipson P, Chen R, Fitzpatrick NK, Damant J, Shipley M, et al Evaluating the quality of research into a single prognostic biomarker: a systematic review and meta-analysis of 83 studies of C-reactive protein in stable coronary artery disease. PLoS Med. 2010;7:e1000286 10.1371/journal.pmed.1000286 20532236 PMC2879408

[19] Don BR, Kaysen G. Serum albumin: relationship to inflammation and nutrition. Semin Dial. 2004;17:432–7. 10.1111/j.0894-0959.2004.17603.x 15660573

[20] Horwich TB, Kalantar-Zadeh K, MacLellan RW, Fonarow GC. Albumin levels predict survival in patients with systolic heart failure. Am Heart J. 2008;155:883–9. 10.1016/j.ahj.2007.11.043 18440336

[21] Fuhrman MP, Charney P, Mueller CM. Hepatic proteins and nutrition assessment. J Am Diet Assoc. 2004;104:1258–64. 10.1016/j.jada.2004.05.213 15281044

[22] Anand IS, Latini R, Florea VG, Kuskowski MA, Rector T, Masson S, et al C-reactive protein in heart failure: prognostic value and the effect of valsartan. Circulation. 2005;112:1428–34. 10.1161/CIRCULATIONAHA.104.508465 16129801

[23] Deswal A, Petersen NJ, Feldman AM, Young JB, White BG, Mann DL. Cytokines and cytokine receptors in advanced heart failure: an analysis of the cytokine database from the Vesnarinone trial (VEST). Circulation. 2001;103:2055–9. 10.1161/01.cir.103.16.2055 11319194

[24] Aukrust P, Ueland T, Müller F, Andreassen AK, Nordøy I, Aas H, et al Elevated circulating levels of C-C chemokines in patients with congestive heart failure. Circulation. 1998;97:1136–43. 10.1161/01.cir.97.12.1136 9537339

[25] Çınar T, Çağdaş M, Rencüzoğulları İ, Karakoyun S, Karabağ Y, Yesin M, et al Prognostic efficacy of C-reactive protein/albumin ratio in ST elevation myocardial infarction. Scand Cardiovasc J. 2019 3 5:1–8. 10.1080/14017431.2019.1590628 30835559

[26] Karabağ Y, Çağdaş M, Rencuzogullari I, Karakoyun S, Artaç İ, İliş D, et al Relationship between C-reactive protein/albumin ratio and coronary artery disease severity in patients with stable angina pectoris. J Clin Lab Anal. 2018;32(7):e22457 10.1002/jcla.22457 29667724 PMC6816976

[27] Libby P, Ridker PM, Maseri A. Inflammation and atherosclerosis. Circulation. 2002;105:1135–43. 10.1161/hc0902.104353 11877368

[28] Libby P, Ridker PM, Hansson GK; Leducq Transatlantic Network on Atherothrombosis. J Am Coll Cardiol. 2009;54:2129–38. 19942084 10.1016/j.jacc.2009.09.009 PMC2834169

[29] Ross R. Atherosclerosis—an inflammatory disease. N Engl J Med. 1999;340:115–26. 10.1056/NEJM199901143400207 9887164

[30] Bustos C, Hernández-Presa MA, Ortego M, Tuñón J, Ortega L, Pérez F, et al HMG-CoA reductase inhibition by atorvastatin reduces neointimal inflammation in a rabbit model of atherosclerosis. J Am Coll Cardiol. 1998;32:2057–64. 9857893 10.1016/s0735-1097(98)00487-2

[31] Aikawa M, Rabkin E, Sugiyama S, Voglic SJ, Fukumoto Y, Furukawa Y, et al An HMG-CoA reductase inhibitor, cerivastatin, suppresses growth of macrophages expressing matrix metalloproteinases and tissue factor in vivo and in vitro. Circulation. 2001;103:276–83. 10.1161/01.cir.103.2.276 11208689

